# Persistent, sterile necrotizing granulomatous dermatitis following treatment of 20-year *Mycobacterium marinum* infection

**DOI:** 10.1016/j.jdcr.2024.08.028

**Published:** 2024-09-16

**Authors:** Eliza Broadbent, Adam M. Spivak, Jacob Kartes, Phillip Lawyer, David A. Wada, Jamie Zussman, Timothy Schmidt

**Affiliations:** a Department of Dermatology, University of Utah School of Medicine, Salt Lake City, Utah; b Monte L. Bean Life Science Museum, Brigham Young University, Provo, Utah

**Keywords:** dermatitis, granulomatous, *Mycobacterium marinum*, persistent, sterile

## Introduction

*Mycobacterium marinum* is a rare cause of human infection, with an annual incidence of 2.7 per 100,000 in the United States.^1^ The slow-growing organism inhabits aqueous environments, infecting injured skin. Sources of exposure include aquariums, contact with marine animals, and salty or brackish water. *M marinum* infection is typically confined to the skin and soft tissues in immunocompetent individuals.^2^ Most incidents (90%) are in the upper extremities.^3^ Poor sensitivity of bacterial culture and lack of a standardized antibiotic regimen make diagnosis and treatment difficult.^4^^,^^5^ We report a case of *M marinum* diagnosed 2 decades after inoculation and an associated granulomatous dermatitis that persisted for months after mycobacteria were eradicated.

## Case report

A 72-year-old man presented to an outside clinic with a tender plaque on his right knee. The lesion arose at the site of a healed abrasion sustained 2 decades previously, during his military service. While at the Chesapeake Bay, he entered the water to save a child from drowning and scraped his knee on a rock. The wound underwent 2 incision and drainage procedures, one a few months after the incident and one several years later, eventually healing to a scaly plaque. The plaque remained approximately the same size, estimated as 4 cm, in the intervening decades, but new tenderness prompted the patient to seek dermatologic consultation. One dermatologist thought the plaque was consistent with psoriasis and prescribed topical and systemic anti-inflammatory medications. The plaque worsened over the next 3 years, and the patient was referred to our care at the University of Utah.

**Fig 1:**
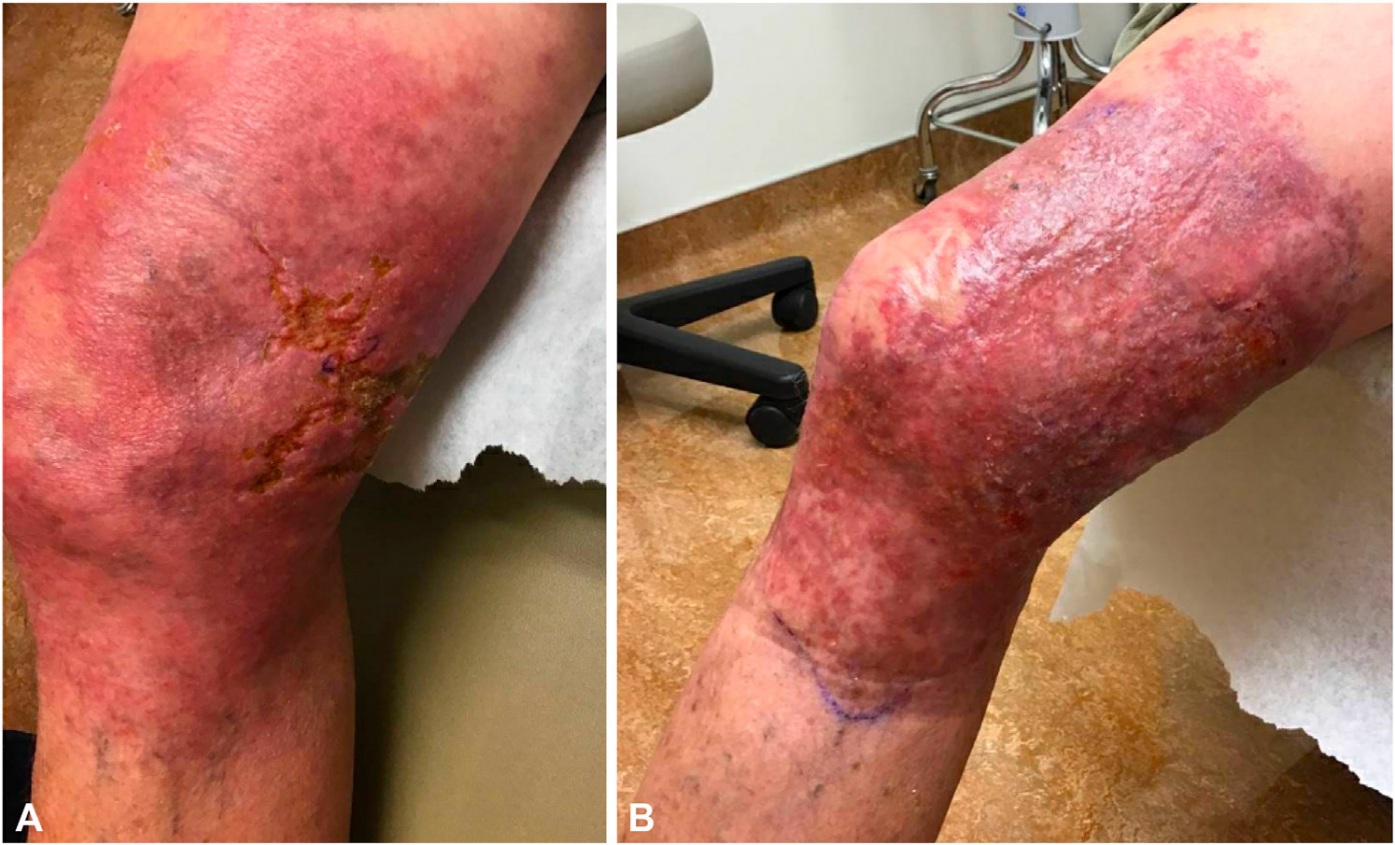
*Mycobacterium marinum* infection featuring a violaceous, ulcerating plaque with vasculitis on the right, distal, medial thigh at presentation (**A**) and 3-month follow-up (**B**).

**Fig 2:**
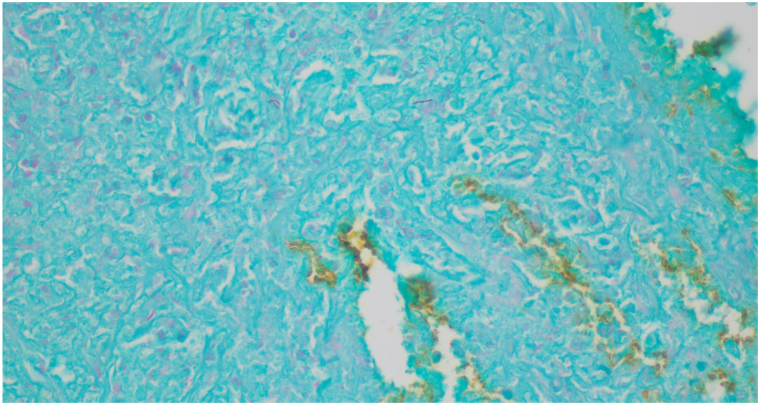
Histopathology from initial skin biopsy with positive AFB staining. *AFB*, Acid-fast bacilli.

Upon presentation, vital signs were normal. The right medial knee and thigh had a tender, indurated, violaceous, ulcerated plaque with irregular borders, approximately 10 cm in diameter (Fig 1, *A*). Our differential diagnoses included infection (bacterial and fungal), leishmaniasis, and granulomatous mycosis fungoides. The patient was empirically started on doxycycline 100 mg twice daily. Punch biopsies were obtained from the ulcer border for bacterial, fungal, and acid-fast bacilli (AFB) culture as well as microbial polymerase chain reaction (PCR) (test performed by the University of Washington). PCR and culture studies demonstrated *M marinum* and *Pseudomonas aeruginosa*. Histopathology with AFB staining was positive (Fig 2). Dermatopathology tissue exam found necrotizing vasculitis of dermal blood vessels with vascular and extravascular granulomas.

The patient’s condition worsened. We consulted our colleagues in infectious disease, who recommended adding levofloxacin, 750 mg daily, for pseudomonal coverage. An magnetic resonance imaging ruled out deep tissue cold abscess or knee joint involvement. At his 2-week follow-up, the patient reported worsening pain with ongoing ulceration. We started tramadol and silver sulfadiazine 1% cream, applied topically every other day. A month later, the plaque was not healing well. Levofloxacin and doxycycline were discontinued. Based on AFB culture and sensitivity results, clarithromycin, 500 mg twice a day, and rifampin, 300 mg twice a day, were initiated. At the patient’s 3-month follow-up, examination revealed some healing but with scattered pustules (Fig 1, *B*). After 6 months of antibiotics, the affected area continued to show signs of inflammation, including a rash of persistent red papules and pustules; however, AFB cultures were negative.

**Fig 3:**
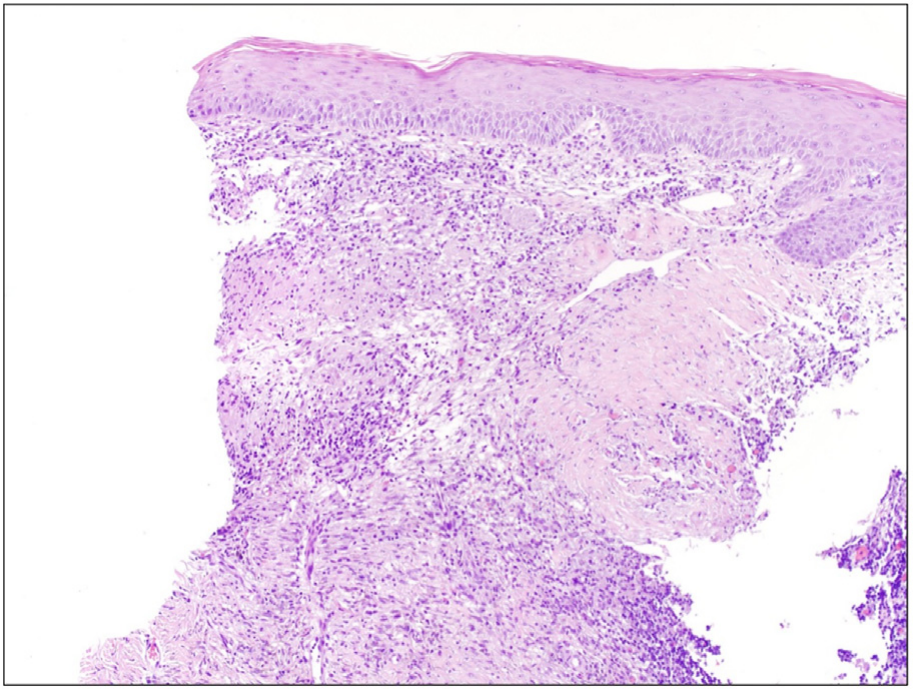
*M marinum* infection resolved with persistent, necrotizing, granulomatous dermatitis seen on histopathology with H&E staining. *H&E*, Hematoxylin and eosin.

**Fig 4:**
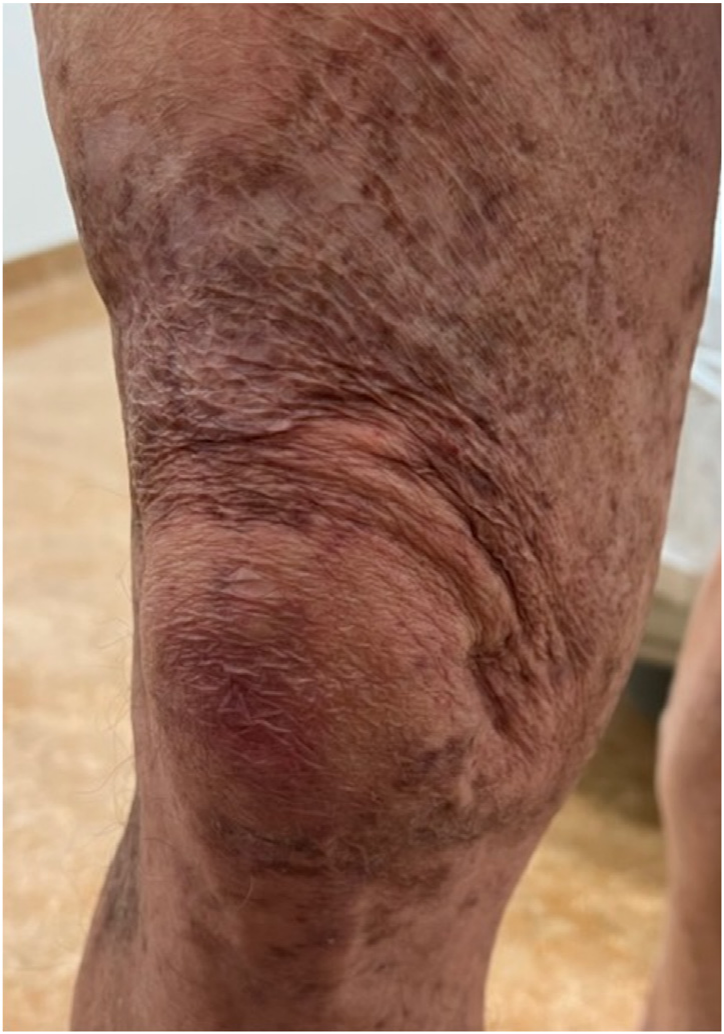
*M marinum* infection resolved with post-inflammatory pigmentation and scarring.

At this point, our differential diagnosis for the rash included an ongoing infection versus a persistent inflammatory dermatosis in the same location despite resolution of infection, such as atypical Sweet syndrome or vasculitis. Punch biopsies from the ulcer border were obtained for hematoxylin and eosin and AFB culture, as well as repeat microbial PCR. Hematoxylin and eosin revealed a necrotizing granulomatous dermatitis highly suspicious for persistent mycobacterial disease (Fig 3). However, culture and PCR were negative, indicating infection resolution. Antibiotics were stopped, and aggressive topical anti-inflammatory treatment was initiated with betamethasone dipropionate augmented 0.05% and tacrolimus 0.1%. The rash gradually improved. One year and 4 months after the patient’s initial presentation at the University of Utah Health, the rash had healed to a scarred, dyspigmented plaque (Fig 4).

## Discussion

*M marinum* is a gram-positive, acid-fast bacillus that causes skin infection in open wounds exposed to contaminated water. Clinical presentation is variable and may feature ulcerations, nodules, and pustules around the site of injury, typically in the upper extremities.^2^^,^^3^ Infection spreads along the path of lymphatic drainage, resulting in a sporotrichoid distribution pattern, and can migrate to deeper structures, causing tenosynovitis, osteomyelitis, or septic arthritis.^4^^,^^6^ Histology findings include granulomatous inflammation and dermal fibrosis.^5^ A review of 63 cases of *M marinum* infection reported a median time of 16 days between inoculation and lesion appearance, with a maximum of 292 days.^3^ In our patient, inoculation with *M marinum* 2 decades prior to presentation initially obscured the connection between exposure and symptoms. Diagnosis is further complicated by the low sensitivity of bacterial culture, which is the principal method used for organism identification.^7^

No standard drug regimen exists for treatment of *M marinum*. Monotherapy is often sufficient for cases of superficial cutaneous infection but is inadequate when deeper structures are involved.^6^ Current guidelines set by the American Thoracic Society and Infectious Diseases Society of America recommend treating with 2 active drugs and continuing therapy for one to 2 months following clinical symptom resolution.^8^ A combination of agents such as clarithromycin, ethambutol, and rifampin has been shown to be effective.^9^ Another study reported an average treatment duration of 25 weeks, suggesting our patient’s prolonged treatment course is not atypical.^7^ Greater depth of infection likely played a role in his case as deeper infections require more lengthy regimens.^6^ However, it is possible that, despite the ongoing rash, the infection had been fully cleared months before we eventually biopsied for microbial PCR.

The pathophysiology of the patient’s persistent inflammation despite infection resolution remains unknown. To our knowledge, no prior studies have reported similar findings post-treatment of *M marinum*. While we do not know exactly when sterilization was achieved, we speculate that either slow bacterial antigen clearance and/or a persistent, localized immunologic memory drove ongoing inflammation and poor wound healing for months after live bacteria were obliterated. Indeed, prior investigation of mycobacteria has shown long latency periods for bacterial digestion, with mycobacteria remaining present in macrophage phagosomes even late in infection.^10^ If inflammation persists at the site of *M marinum* infection for more than 3 months despite appropriate antibiotics, repeated biopsies for culture and microbial PCR may allow for an earlier transition from antibiotics to an anti-inflammatory treatment.

## Conflicts of interest

None disclosed.
